# Supplementary material for: Are carbon emissions trading and green financial instruments synergistic? -Comprehensive quantitative research based on content analysis
Source: PLoS One. 2024 Mar 7;19(3):e0298601. doi: 10.1371/journal.pone.0298601 (PMC10919601; doi:10.1371/journal.pone.0298601)
Supplement: S1 Data — (ZIP) [file pone.0298601.s001.zip › Data/地级市Z_F工作报告.zip!/╣π╢1⁄2/╔╟╬▓╩╨/└┤╫╘╣1⁄2╓┌║┼í╛┬φ┐╦╩2╛▌═°í┐_2010.docx]

各位代表：

　　我代表市人民政府向大会作政府工作报告，请予审议，并请政协各位委员和其他列席人员提出意见。

2009年工作回顾

　　2009年是新世纪以来我国经济发展最为困难的一年，也是汕尾备受考验的一年。在国际金融危机突如其来，世界经济局势急剧下滑，全省各地纷纷减速的严重局面之下，正在迎头赶上的汕尾受到严峻的挑战。面对复杂的形势，我市在党中央、国务院和省委、省政府的正确领导下，不畏困难，咬定牙关，共克时艰，坚持深入贯彻落实科学发展观，认真贯彻落实党的十七届三中、四中全会、省委十届四次、五次全会和市委五届五次、六次全会精神，以积极进取、危中取机的姿态应对金融危机，大胆提出"冲过去"口号，大力实施"弯道超车"战略，以"四推进一保持"为工作主线，贯彻"四再"理念（再苦也要上大项目、再低也要转型升级、再穷也要保护环境、再难也要树立形象），认真落实"五补"措施（出口不足投资补、外资不足内资补、生产不足流通补、产能不足管理补、硬件不足软件补），突出抓好"五大战役"（重点项目、交通建设、"百里海堤"、园区建设和民生实事），实行责任榜制度，狠抓工作落实，有力地促进了经济社会持续较快发展。全市主要经济指标逆势飘红，增幅位居全省前列，排名再创历史新高，"两个确保"如期完成，"弯道超车"不负所望，"活力汕尾、人文汕尾、和谐汕尾、清新汕尾"建设加快推进。概括起来，主要体现在以下九个方面：

　　一、经济发展逆势飘红，综合实力不断增强

　　经济运行保持快速增长势头，增长速度逐季加快。据测算，全年全市实现地区生产总值396亿元，比上年增长16.3%；全社会工业总产值579.2亿元,增长20%，其中规模以上工业总产值318.8亿元,增长26.4%；全社会固定资产投资289.4亿元，增长38%；实际利用外资2.8亿美元，增长9.6%；外贸出口总值10亿美元，下降7.8%；财政一般预算收入19.71亿元，增长25.16%；社会消费品零售总额288.1亿元，增长24.1%；农业总产值110亿元，增长6.5%；市区居民人均可支配收入12812元, 增长15.8%；农民人均纯收入5372元，增长12.2%。"十一五"规划实施取得了阶段性成效，主要发展指标完成情况良好。全社会固定资产投资、地方财政一般预算收入、社会消费品零售总额、外商直接投资、新型农村合作医疗覆盖率、人口自然增长率等11项指标提前一年完成。

　　二、应对危机有为有效，产业体系不断完善

　　积极贯彻落实中央应对国际金融危机一揽子计划，落实"五补"措施，加大中小企业扶持力度，促进企业转型升级，努力化解外部需求不足和经济效益下滑等新挑战新考验，经济效益明显回升，产业结构不断优化。三次产业结构由2008年的18：46.4：35.6调整为16.5：46：37.5，第三产业比重进一步提高；工业结构进一步调整，工业规模化程度明显提高，规模以上工业总产值占工业总产值的比重上升55%；新增工业企业127家，其中规模以上工业企业40家；全社会用电量26.7亿千瓦时，增长9.2%，其中工业用电量13.8亿千瓦时，增长13.2%，工业用电需求增幅领先全省。食品制造、纺织服装、珠宝首饰等传统产业景气度保持高位增长，其中食品制造、珠宝首饰行业增幅均超过50%。工业经济效益明显好转，规模以上工业利润总额11.1亿元，比增98%。争取中央预算内和新增中央投资项目96个，得到中央和省专项补助资金6.2亿元，带动地方投资11亿元。

　　三、重点项目拉动明显，城市建设步伐加快

　　2009年列入省重点项目9个，全年完成投资53.1亿元，完成年度计划110.8%；我市安排23个重点建设项目全部开工建设，全年完成投资57.8亿元。厦深铁路汕尾段累计完成投资超过28亿元。汕尾电厂一期3#、4#机组完成投资15.7亿元，超额完成年度任务。陆丰甲湖湾风电场已建成投产。华润海丰电厂已获得国家能源局批准开展前期工作。陆丰核电站进厂道路已竣工通过验收。百里海堤、荒山绿化、污水处理、垃圾处理、饮水安全等"五大生态安全工程"建设加快推进。海堤建设已完成89公里。汕尾市区（西区）和陆河县城两个污水处理厂已建成运行。深圳（汕尾）产业转移工业园建设全面启动，首期规划面积扩大到13.08平方公里，完成了工业园的总体规划、环评等工作，全年完成投资1.75亿元。中国泛华集团深圳分公司以B.T合作方式，投入30亿元建设园区基础设施和公共设施。各县（市、区）的重点项目有效推进。城市基础设施不断完善。香洲东路、金湖路等市区主要道路已完成主体工程。碧桂园一期已顺利开盘。全市行政村道全面竣工，879个村（社区）100%通硬底化道路。"十项民生实事"全面开工，其中马宫街道居民饮水工程、市儿童福利院、市妇幼保健院、廉租房建设工程、68.5万人农村饮水安全工程、社会保险市级统筹等6个项目已全面完成。城市规划日趋完善，"四规合一"规划修编有序推进，城市管理不断加强，城市品位快速提升，市容市貌明显改善。

　　四、"三农"工作扎实推进，效益农业初见成效

　　农业经济快速发展，新农村建设步伐加快。全市完成农业总产值110亿元，比增6.5%。粮食生产喜获丰收，全年粮食总产量达到51.3万吨，增长21.1%，超额完成了省粮食工作考评指标。农业产业化、基地化、规模化进程加快，农业龙头企业40家，带动农户4.96万户。农民专业合作组织83家，带动农户2.9万户。渔业稳步发展，水产品总产量54.9万吨，增长2.0%；渔业总产值63.2亿元,增长7.1%。强农惠农政策进一步落实，现代标准化农田建设步伐加快，农业综合生产能力明显提高。水利工程建设加快推进，全年完成投资5.4亿元，抵御自然灾害的能力不断增强。"规划到户、责任到人"扶贫开发工作全面铺开，农村饮水安全得到有效解决，农村面貌明显改善。造林绿化工作稳步推进，陆河县创建"林业生态县"已通过省验收，海丰县联安镇被评为"中国最佳绿色生态名镇"。

　　五、财税工作成绩突出，信贷投放力度加大

　　财政收入继续保持大幅增长，全市完成财政一般预算收入19.71亿元，增长25.16%，实现了三年翻一番的预期目标，甩掉全省最后的"尾巴"，排名晋前两位。收入结构好转，税收收入增长30.7%，增幅居全省第一，高出财政一般预算收入5.5个百分点，非税比降低2个百分点。财政收支平衡，略有节余。年末全市金融机构各项人民币存款余额276.9亿元，比年初增长19.7%；金融机构各项人民币贷款余额97.4亿元，比年初增长49.2 %。全市金融机构实现全面盈利，贷款余额增长速度明显加快。

　　六、招商引资形成高潮，外经外贸企稳回升

　　面对国际金融危机的冲击，外向型经济经受考验，保持稳步发展。实际吸收外商直接投资2.8亿美元，增长9.6%。出口下滑势头得到扭转，下半年出口规模扩大，降幅明显收窄。全市新增外贸经营主体26家。海崇畜牧、国泰水产、信利电子、德昌电子等4家企业被评为"重点培育和发展的广东省出口名牌"。设立新南方综合保税仓，填补了我市海关特殊监管区的空白。汕尾港红海湾港区获国务院批准对外开放。通过举办产业转移招商会、光彩事业汕尾行、国庆前"开工一批、剪彩一批"活动，参与粤东侨博会、省组织的各类招商会等，开工、签约一批项目，合作总额达700多亿元，其中信利4.5代TFT、陆丰内湖农批、海丰陆港农业、华孚冻库、中恒药业、恒海水产、国电新能源、恒大商贸城、天洋太阳能光伏、华润水泥、鮜门再生能源博物馆等优质、高端项目为我市提供了强力的投资储备。

　　七、消费市场持续畅旺，商贸旅游日显繁荣

　　全年完成社会消费品零售总额288.1亿元，增长24.1％。新增个体工商户8682户，增长52%；新增私营企业441家，增长71%。"家电下乡"活动和"万村千乡"市场建设工程扎实推进，新建、改造农家店50家。旅游业加快发展，旅游总收入25.5亿元，增长11.3%；接待游客405.6万人次，增长20.6%。房地产业异军突起，房地产销售总额21.1亿元，增长217.3%。

　　八、和谐汕尾全面推进，社会事业协调发展

　　大力推进平安工程建设，开展"强综治、创平安、促维稳"先行创建活动并率先在全市铺开，海丰维稳综治中心试点经验在全省推广。各项严打整治活动和社会矛盾排查化解活动深入开展，刑事立案数、每万人刑事发案数均排在全省末位，全年全市共立各类刑事案件2522宗，破案927宗，破案数上升2.5%,破案率上升6.6%,立案数下降14.6%。整治碣石非法加工经营旧服装等专项整治活动取得明显成效。安全生产"四项指标"全面下降，总体测评优良。产品质量及食品安全的监督管理不断强化。甲流疫情得到有效控制。以县委书记为主体的大接访活动深入开展，信访工作长效机制不断加强。社会保险覆盖面不断扩大，全市征收各项社保费5.8亿元，增长21.5%。各项社保待遇不断提高，企业离退休人员养老金等按时足额发放。城镇居民医疗保险覆盖面不断扩大，陆河县农村养老保险试点工作顺利推进。新农合参合率达99.1%。全年全市新增城镇就业4万人，转移农村劳动力5.3万人，城镇登记失业率为2.76%。城乡低保"扩面提标"，社会救助体系进一步完善。双拥工作大力推进，优抚工作成效显著。教育水平明显提高，高中阶段教育稳步推进。实行"议教"制度和市领导挂扶筹款办学机制，市委书记、市长向各县（市、区）书记、县(市、区)长发出"教育一封信"，有效推动了全市教育事业加快发展。初中毕业生升学率提高18个百分点，高中毛入学率提高11个百分点。开建华师附中汕尾学校和汕尾理工职中。2009年高考本科上线人数突破4000人。自主创新能力不断增强，预计全年全市专利申请量260件，增长58%。人口计生工作取得新成绩，人口出生率10.57&permil;，人口自然增长率6.06&permil;，计划生育率为93.75%，同比上升4.39个百分点。人才工作稳步推进。精神文明建设扎实开展。文化工作取得新进展。亚运会汕尾赛区筹备等体育工作卓有成效。殡葬管理工作进一步加强。审计、统计、法制、国土、人事、人防、打私、国防、气象、防震、外事、侨务、台务、物价、司法、扶贫、环保、民族宗教、档案修志、老龄、残联、妇女儿童等工作取得新成绩。

　　九、政务环境不断改善，政府执行力逐步提高

　　自觉接受人大监督、政协民主监督、新闻舆论监督和公众监督，积极办理人大代表议案、建议和政协提案，政府工作法制水平不断提高，市审计局被授予"全国审计系统先进集体"称号。落实市领导分工负责重点工作责任榜，深入开展"形象年"建设活动和"行风热线"、"民主评议公务"活动，积极开展对各级机关作风的明查暗访工作，加大对重点项目和重要工作的督查督办力度，"权威、责任、效能、廉洁"政府建设不断加强，政务环境不断优化。"金钥匙"保姆式服务工程、一站式政务服务、订单式政务服务三种模式加强无缝覆盖，为投资者提供"亲商"服务环境。工作作风不断转变，以市委、市政府名义召开的全市性会议同比减少49.1%，全市公务接待费用同比减少26.3%。工作措施不断创新，采用现场会解决实际问题的办法，全年共召开经济工作、教育、财税、公路、园区、减排、节能、电网、外经贸等12个现场会，有效解决了村道建设、饮水改造、水库除险、污水处理、高中教育、节能减排、园区建设、财税增收等一系列突出问题。

　　各位代表，过去一年取得的成绩来自不易，是在省委、省政府和市委的正确领导下，在市人大及其常委会、市政协的大力支持下，全市人民共克时艰、团结拼搏的结果。在此，我谨代表市人民政府，向全市广大工人、农民、知识分子、干部职工，向驻汕人民解放军、武警官兵和各民主党派、人民团体、社会各界人士表示崇高的敬意！向所有关心支持我市发展的港澳同胞、台湾同胞、海外侨胞、国际友人以及外地朋友表示衷心的感谢！

　　回眸2009年,我们既取得了显著的成果，又积累了在复杂经济环境中推动经济社会又好又快发展的重要经验。一是必须审时度势，临危不惧，善于准确判断形势，善于抢占先机，敢于在复杂环境和重要转折点推进事业发展；二是必须坚持解放思想、实事求是、改革创新，把上级的决策部署与我市实际紧密结合起来，以科学发展谋后来居上；三是必须坚持发展第一要务，用加快发展的办法解决前进道路上遇到的困难和问题，用加快发展的成果改善民生，惠及广大群众；四是必须激发发展活力，坚持自主创新，推动产业转型升级，着力提高经济增长质量和效益。

　　在肯定成绩的同时，我们也清醒地认识到，我市经济社会发展仍存在一些问题和困难，主要是：国际经济环境不确定不稳定因素还很多；我市经济基础薄弱，经济总量小，去年虽然大幅增长，占省比重突破1%，但仍块小体弱；工业化水平和产业集聚度低，规模以上工业增加值依然是经济领域最大的落后尾巴；对外贸易形势依然严峻，外部需求依然疲弱，出口短期内增长压力巨大；新增产能的项目不多，特别是生产性大项目少，支撑经济快速发展的生产能力不够强劲；软环境仍待进一步改善，干部的执行力有待提高；计生、教育、卫生、殡改、社会保障、社会治安等方面还存在很多问题有待解决。对此，我们必须高度重视，切实采取措施加以解决。

2010年工作安排

　　2010年是我市实施"十一五"规划的收帐年，也是我市冲刺人均GDP2000美元的跨越年，办好亚运会帆船赛、亚洲帆船锦标赛、惠州同乡恳亲会的形象年，更是我市实现"五年大变化"目标的关键之年。做好今年全市经济社会各项工作，对于进一步有效应对国际金融危机，巩固经济快速发展势头，实现汕尾科学发展、跨越发展至关重要。

　　综观国际国内形势，我市今年经济发展总体环境将明显好于去年。从外部环境看，当前国际金融市场渐趋稳定，功能正在恢复，主要经济体经济出现好转迹象，一些新兴经济体和发展中国家经济增速加快，世界经济有望恢复性增长。国内经济运行的积极因素不断增加，经济回升向好的基础逐步巩固，市场信心明显增强，扩大内需和改善民生等政策效应将继续显现，企业适应市场变化的能力和竞争力不断增强。中央决定继续实施积极的财政政策和适度宽松的货币政策，不断充实完善应对国际金融危机冲击的一揽子计划和政策措施，以及《珠江三角洲地区改革发展规划纲要》、《关于促进粤东地区实现"五年大变化"的指导意见》等政策的出台实施，"双转移"战略的全面推进，都为我市加快发展提供了难得的发展机遇。同时，我市经过连续六年快速发展，经济发展的内动力日益增强，经济合作的外推力不断增长，经济风险的免疫力日渐形成，经济社会的互动力逐步改善，必将为下一步又好又快发展创造有利条件。只要我们把握机遇，科学谋划，真抓实干，我市今年经济社会平稳较快发展，是完全可以实现的。

但我们也要清醒地看到，今年我市经济社会各项工作仍将继续受到金融危机的冲击和影响，外部不确定因素依然存在，加快发展将面临不少的压力和困难。对此，我们既要充满信心，乘势而上加快发展，又要增强忧患意识，未雨绸缪谋划发展；既要把困难估计得更充分一些，把应对措施考虑得更周密一些，又要注重从变化的形势中捕捉和把握难得的发展机遇，发现和培育有利因素，统筹市内市外两个大局，扎扎实实把各项工作做好。

　　今年工作的总体要求是：以邓小平理论和"三个代表"重要思想为指导，深入贯彻落实科学发展观，全面贯彻党的十七届四中全会、中央经济工作会议、胡锦涛总书记视察广东重要讲话精神和省委十届六次全会、粤东地区工作会议、粤东地区（汕尾）现场会及市委五届七次全会精神，以"五年大变化"和全面"砍掉落后尾巴"为目标，贯彻"四再"理念，坚定不移实施"四推进一保持"，以产业集聚力促现代产业体系，以宜居城乡力促扩大内需，全力促进经济发展方式转变，努力实现经济社会又好又快发展。

　　今年全市经济社会发展的主要预期目标是：生产总值增长18%；工业总产值增长26%，其中规模以上工业总产值增长40%；规模以上工业增加值增长38%；农业总产值增长6.5%；全社会固定资产投资增长25%；社会消费品零售总额增长20%；外贸出口总值增长12%；外商直接投资增长16%；地方财政一般预算收入增长25%；城镇居民人均可支配收入增长10%；农民人均纯收入增长9%；居民消费价格涨幅控制在3%左右；城镇登记失业率控制在3%以内；人口自然增长率控制在6.13&permil;以内；万元GDP能耗下降、二氧化硫和化学需氧量排放总量减排实现"十一五"目标。

　　实现今年经济社会发展目标，必须突出重点，统筹兼顾，在"十个加快"上狠下功夫：

　　一、以推进产业集聚为龙头，加快建设现代产业体系

　　做大规模以上工业。把今年作为"工业年"，落实领导抓工业责任制，加大工业企业清理清查力度，进一步规范工业企业管理，力争增加规模以上工业企业80家。落实责任、重点跟踪，抓好投资500万元以上生产性工业项目上马建设，重点抓好信利4.5代TFT、中恒药业、恒海水产、华润水泥、现代物流、3.5代平板、天洋太阳能光伏、国电新能源、游艇制造、再生能源示范区等10个30亿元以上项目，通过争取早日落户、上马、开工，集聚现代产业，力促我市现代产业体系的形成。

　　做优电子信息产业。依托信利、德昌等龙头企业，以TFT、OLED、二极管和三极管等产品为龙头，大力引进上下游产业，延长和完善电子信息产业链，做大做强电子信息产业基地。把深圳（汕尾）产业转移工业园东片区建设成为具有较大影响力的省级电子信息产业园，力争全市电子信息产业规模跃上新台阶，努力打造我市重要的支柱产业。

　　做强电力能源产业。加快推进汕尾电厂一期3#、4#机组、陆丰核电站、华润海丰电厂、宝新能源、甲湖湾新能源基地等电力能源项目建设，力争华润海丰电厂、陆丰核电站下半年开工建设，确保汕尾电厂一期3#、4#机组年底建成投产，加快发展海上风电项目，力促太阳能光伏等新能源、再生能源项目落户汕尾，支持山区小水电开发建设，大力推进太阳能等可再生能源项目前期工作，打造电力能源基地。

　　做大石油化工产业。充分发挥区位优势，主动承接近邻炼化基地下游产业，发展配套产业和开发下游产品，加快海丰小漠华城石化基地、珠三角成品油管道二期工程汕尾段及分输站、天然气输气管道、明发油脂化工的建设，做大石化产业。

　　做新传统产业。积极实施名牌带动战略，推进自主创新，调整优化产业结构，壮大纺织服装、食品制造、金银珠宝首饰加工、圣诞工艺品等传统支柱产业，提升"汕尾制造"竞争力。鼓励工业企业创建研发中心，形成一批拥有自主知识产权的技术和产品。大力推进企业技术改造，推动技术装备提档升级、产品更新换代。扶持一批成长型、税源型、就业型中小企业成长，提高规模以上工业企业在传统产业的比重。

　　二、以重点项目建设为引擎，加快拉动经济发展

　　加快重点项目建设。2010年省安排我市重点项目14个（包括2个预备项目），年投资总额243亿元；我市初步安排市重点建设项目36个，总投资910.8亿元，年度投资102.1亿元；预备项目32项。重点抓好信利4.5代TFT、汕尾电厂一期3#、4#机组、华润海丰电厂、陆丰核电站首期工程、厦深铁路汕尾段、省道241线汕尾至遮浪段扩建、深圳（汕尾）产业转移工业园、市区红海西路、金湖路（碧桂园段）、工业大道东段、市区防洪（潮）工程、华师附中汕尾学校、市区碧桂园商住房工程等重点项目建设。做好陆丰甲湖风电场二期工程、华润海丰水泥厂等预备项目前期服务工作，力争尽早开工建设。加强项目策划、储备和申报，力争更多项目进入国家和省专项规划，获得政策、资金和项目布点支持。

　　加快交通大网络建设。按照"东联西融打造桥头堡"的总体思路，加快推进"两网一港"（公路网、铁路网、海港）规划建设，加强交通基础设施建设，逐步形成贯通珠三角和海西经济区、接轨长三角、沟通南北的高速公路、铁路网和海上运输通道。重点加快推进厦深铁路汕尾段及配套站场建设，争取福建漳州至广西玉林高速公路汕尾段尽快动工建设，抓好国道324线汕尾段、汕尾至遮浪公路等国省道公路建设。全力做好广州至汕尾铁路、龙川至汕尾铁路、兴宁至汕尾高速公路汕尾段的前期工作。加快完成《汕尾港总体规划》修编工作，修建海丰县小漠中石化码头，加快推进陆丰甲湖湾发电厂码头、华润海丰电厂配套码头、汕尾港航道浚深工程等项目建设，提高汕尾港通航能力和装卸能力，力争港口吞吐量达到500万吨，集装箱吞吐量突破3万个标准箱。

　　突出抓好园区建设。全面落实《纲要》提出的规划部署、重点任务和政策措施，主动融入珠三角，当好"桥头堡"。 着力提高与深圳的合作层次，发挥"三基地"、"三经济带"的保障、带动作用，打造与珠三角产业对接、供应保障、空间互补的基地经济、海岸经济、配套经济，在发展电子信息、电力能源、石油化工、先进制造、海洋技术、滨海旅游、生态农业、港口物流等方面，成为珠三角的产业基地。全力推进"双转移"工作，落实好省"双转移"考核制度。立足深汕特别合作区200平方公里合作，通过不懈努力，把合作区建设成为广东省乃至全国统筹区域发展的示范区、区域合作机制创新试验区、粤东大型先进产业园区和现代化综合性新城区。全力加快首期13.08平方公里的深圳（汕尾）产业转移工业园建设，加快推进与泛华集团深圳分公司的B.T项目合作，今年要全面完成园区基础设施建设，掀起园区招商引资热潮，促使入园项目快出产能。健全园区管理机构，不断完善园区运作机制、管理机制、监督机制，争取尽快建成省高端产业转移工业园。同时做好申报省级高新技术开发区的工作。继续推进红海湾经济开发区、海丰经济开发区、陆丰东海经济开发区、星都经济开发区的建设。

　　三、以宜居城乡建设为载体，加快发展宜居宜业滨海新城

　　按照宜居城乡要求进一步完善城乡规划。以"宜居之城、品质之都"为总目标，以"海在城中、城在山间"为主线，以"四规合一"规划修编统筹宜居城乡建设规划。做好城市总体规划、产业布局、土地利用、环保等规划修编扫尾工作。围绕打造"一(中)心一轴、一带三片(区)、多组团"的城市发展建设总体空间结构，推动汕尾市区适度东拓，建设汕尾新中轴线。

　　着力推进城乡基础设施建设。抓好2009年十项民心工程扫尾工作。推进市区火车站片区基础设施建设，推进品清湖疏浚和沿岸综合开发项目建设。以抓好第16届亚运会海上项目汕尾分赛区基础设施建设为契机，以市区--红海湾的绿道规划建设为先行试点，大力推进绿道网建设，逐步与珠三角绿道网对接。有序推进旧城镇、旧厂房、旧村庄的"三旧"改造工作。加快推进电网规划建设。加快中国（汕尾）民间艺术馆、市规划博物馆以及农家书屋等公益性文化基础设施建设。以改善城镇中低收入家庭住房条件为重点，加快推进廉租房和经济适用房建设，2010年解决城镇低收入家庭住房困难340户。

　　着力建设生态文明环境。以建设"五大生态安全工程"为龙头，加快生态安全基础设施建设；严格环保市场准入，从源头上控制污染；加大力度完成六个污水处理厂建设扫尾工作，确保一季度投产通水，启动建设全市生活垃圾无害化处理厂；加大力度落实节能措施，确保节能工作合格；加强对大湖鸟岛、公平自然生态保护区、陆河红椎林保护区的保护，加快建设陆河生态文明县；加强造林绿化、水土保护；大力发展循环经济，推行清洁生产，广泛开展节水节地节材活动；严格控制建设用地规模，严格查处违规用地，提高土地利用效率。

　　四、以现代农业建设为平台，加快推进"三农"工作

　　提升现代农业发展水平。构建以质量效益为重心的现代农业体系，在稳定粮食生产的基础上，全力建设好农业种养"十大基地"和"五大园区"。培育发展一批农业龙头企业和专业合作社组织。加快发展特色农业，加快农业产业化进程，提升全市农业现代化水平。加强水利基础设施和现代标准农田建设，按省的要求，确保今年全面完成防灾减灾和病险水库除险加固工程建设。

　　加快发展现代渔业。以汕尾中心渔港建设为重点，全面加快全市12个渔港及其商贸配套设施建设，打造海产品物流商贸中心；大力发展海洋捕捞业和水产品深加工业，加快水产养殖"十大基地"建设，提高海洋渔业产业化水平，打造海洋经济支柱产业。

　　拓宽农民增收渠道。落实好国家各项强农惠农政策，促进农民政策性增收。挖掘农业内部增收潜力,引导农民发展订单农业和旅游观光农业，创新农业经营方式，探索建立农民和企业利益联结机制。加大农业生产技能和就业技能培训力度，鼓励就近就业和返乡创业；加快各县（市、区）综合性职业培训基地建设，实施"一户一技能"计划，开展针对性的"订单式"培训，深化与珠三角地区劳务合作机制，促进农村劳动力转移就业。

　　大力发展农村公共事业。加快农村基础设施建设，完善村庄道路、供排水、沼气等生产生活设施，发动群众整治村庄环境，推进社会主义新农村建设。加大扶贫开发力度，全力抓好 "规划到户、责任到人"扶贫开发工作，用两年的时间，完成249个村3.6万户17.1万人脱贫。

　　五、以招商引资为突破口，加快推进外经贸工作

　　着力抓好招商引资。完善招商引资激励机制和责任机制，创新走出去、请进来的招商途径，运用以商引商、以侨引商、敲门招商、产业招商等方式招商，引导外资投向高端制造、高新技术产业、新能源、节能环保和现代服务业等领域。突出重点办好招商活动，积极引进符合产业规划、具备产业带动效应项目，吸引世界500强、中国500强企业和国有、民营大中型企业投资。

　　着力改善投资环境。整顿完善市行政服务中心，继续完善"金钥匙"保姆式服务工程、一站式政务服务、订单式政务服务，确保招商项目落地生根、开花结果，变地理区位优势为真正的营商优势。加强对全市招商引资的统筹协调，形成"人人都是投资环境，招商引资人人有责"的氛围，综合打造"安商、亲商、稳商、富商"的服务环境。

　　着力抓好外贸出口。用好用足中央、省一系列扶持政策，发挥部门协作效应，提高通关、质检、退税、外汇管理等环节的便利化水平，营造"大通关"环境；畅通出口渠道，努力促进出口稳步回升，鼓励和支持企业参加广交会等国内外知名展览会，帮助企业开拓国际市场，提高出口产品档次、附加值和竞争力。落实工作责任制，组成"金钥匙"保姆式服务组，由市领导带队上门服务，包干做好重点项目、重点客户一对一服务工作，打好外贸工作翻身战。

　　促进加工贸易转型升级。鼓励企业设立研究开发中心，发展自主品牌，优化结构体系，着力抓好内外销市场的拓展，延伸加工贸易产业链，不断推进加工贸易企业加快销售市场转型、生产方式转型、产地转型和发展方向转型，实现发展阶段升级、产业结构升级、产品结构升级和集聚配套升级。

　　六、以继续扩大内需为契机，加快发展第三产业

　　加快发展现代服务业。积极发展为制造业配套的金融、现代物流、科技服务、信息服务、商务服务、市场集群六大生产性服务业。加快发展信息服务业，规范发展专业服务业，培育发展新兴服务业。做大珠宝、金银首饰、服装等专业市场和大型农产品、水产品批发中心。

　　加快发展港口物流业。以国务院批准汕尾港红海湾港区对外开放为契机，加强汕尾新港区岸线、前沿海域、后方陆域的规划管理。做好临港产业集中区、工业园区、物流园区控制性规划，争取海洋功能区划获批。加快港区交通基础设施建设，打造通往内地便捷、快速、通畅的出海通道。努力拓展腹地、延伸辐射，发挥港口对区域经济的带动作用。加快推进汕尾港及新港区物流园、深圳（汕尾）转移工业园物流中心和陆丰内湖农副产品批发市场建设，努力把汕尾新港区建设成为国家一级一类国际港口，倾力打造珠三角地区新的物流基地。

　　加快发展滨海旅游业。全面实施《汕尾市旅游发展总体规划》，整合"红、蓝、绿、古"四色旅游资源。全面加强旅游基础设施建设，加大旅游宣传力度，提升与珠三角地区和海峡西岸经济区旅游合作层次，繁荣区域旅游市场，推动汕尾旅游由观光型向复合型转变，由过境地向目的地转变。不断完善景区配套设施，着力打造滨海度假游、生态绿色游、民俗风情游、红色教育游、古迹观光游等旅游品牌，抓好红宫红场、凤山祖庙旅游区创建国家3A级景区工作。加强旅行社、星级饭店标准化管理，营造良好的旅游发展环境。

　　继续扩大居民消费需求。进一步落实国家关于增加城乡居民收入的各项政策，提高居民特别是低收入群众消费能力。重点培育发展房地产开发市场，走规模开发、集约经济、品牌打造、特色城市的房地产发展道路，推动房地产消费形成高潮，拉动内需促增长。实施"万村千乡"市场工程，进一步做好家电、汽车、摩托车下乡工作，落实家电和汽车以旧换新政策，落实农机购置补贴政策。加强消费者权益保护，优化消费环境，增强消费信心。

　　七、以提高经济社会发展保障能力为目的，加快做大财政蛋糕

　　强化财税征管。落实领导抓税收责任分工，完善每月一次财税工作分析会和联席会议等工作机制，充分发挥各级党政以及各职能部门协税护税积极性，落实收入目标监控制度及各项激励制度。加强重点税种、重点税源企业和重点项目管理；加强经济户口清理和纳税评估，争取在税源挖潜堵漏上取得新突破；坚持依法治税，加大税收稽查力度，严厉打击各种偷、逃、骗税行为。加强税法宣传，优化纳税服务，提高依法纳税遵从度。加快建立国有资本经营预算制度，规范政府资源有偿使用收入管理。

　　提升理财水平。充分发挥财政资金"四两拔千斤"效应，努力提高资金使用效益。各金融机构要努力增加金融有效投入。充分利用国家和省财政政策实施重点，加大对民生领域和社会事业的支持保障力度，加强对"三农"、科技、教育、卫生、文化、社会保障、保障性住房、节能环保等方面和中小企业、居民消费的支持力度。整合财政性引导资金，大力扶持一批在建重点项目。

　　防范化解财政和地方金融风险。依法规范政府投融资行为，完善举债和担保制度，建立更多的担保公司和中介机构，构建中小企业融资平台。做好财政欠债清理和银行业不良贷款清收处理，防范和化解银行业风险，加大反假币工作力度，维护财政金融安全。

　　八、以创新体制机制为动力，加快推进重点领域改革

　　坚持用改革、开放、创新的办法破解发展难题，激发发展活力，增创发展优势，建立健全有利于科学发展的体制机制。深化行政管理体制改革，按照省的部署，完成市、县政府机构改革。扎实推进富县强镇事权改革，大力简政放权,切实转变县镇政府职能,建立责权一致、分工合理、决策科学、执行顺畅的行政管理体制，增强县镇活力。进一步推动政府职能转变，深化行政审批和投资制度改革，减少和规范行政审批，最大限度地维护企业投资主体地位。完善农村土地经营制度改革，推动土地有序流转。推进集体林权体制改革。积极配合开展"省直管县"财政体制改革。加快国有资产管理体制改革，引导国有企业面向市场进行改制重组，逐步化解历史包袱。加快推进文化体制改革。深化医药卫生体制改革，促进基本公共卫生服务均等化，建立覆盖城乡居民的基本医疗卫生制度。

　　九、以保障和改善民生为目标，加快推进社会各项事业发展

　　强化社会保障工作。进一步落实就业扶持政策，稳定与扩大就业，引导和促进劳动密集型企业、中小企业、民营经济、各种服务业加快发展，积极鼓励和支持劳动者自主创业和自谋职业；加强职业技能培训和公共就业服务，加强对就业困难人员和零就业家庭的就业援助。完善社会保险市级统筹，逐步提高社会保障水平。继续推进社会保险扩面征缴，重点抓好民营企业、外资企业等非公有制企业和灵活就业人员的社会保险扩面工作；确保企业离退休人员养老金等按时足额发放。巩固和扩大城镇居民医保，进一步解决困难企业退休人员参加医疗保险问题，全面开展农村新型养老保险工作，提高社会保障覆盖率。做好优抚安置工作。

　　优先发展教育事业。以创建"教育强县、教育强镇"为目标，优先发展教育。完善"议教"制度，解决教育发展瓶颈问题。鼓励和规范民办教育，发展远程教育和继续教育，加快推进教育现代化。落实筹资优先、政策优惠、教师优待、学位优良的"四优"原则，促进义务教育均衡发展。进一步加快高中阶段教育发展步伐，大力发展中职教育，推进示范性普通高中和重点中职学校建设，大办教育园区，推进高中阶段教育集约化发展。重点抓好华师附中汕尾学校和汕尾理工职业学校建设，提升发展好汕尾职业技术学院，力争开建一所民办大学。以提升高中、初中、小学入学率、升学率、就业率为主线，认真做好"防流控辍"工作。实施中小学校舍安全工程，大力改善办学条件。加强校长和教师两支队伍建设，切实解决中小学代课教师和教师工资福利待遇问题。

　　全面发展社会事业。健全完善公共卫生服务体系，提高卫生应急和重大疾病防控能力，重点抓好甲型H1N1流感、人禽流感等重大传染病的防控工作。加快推进农村三级卫生服务网络和城市社区卫生服务中心建设，建设市中医院。提高新型农村合作医疗管理和保障水平，全面加强农村卫生工作。推进文化体育基础设施建设，加强文化遗产的保护和开发利用，扩大"中国民间文化艺术之乡"影响力。集中力量办好第14届亚洲帆船锦标赛、第16届亚运会帆船赛和第七届世界惠州同乡恳亲会。继续完善计生层级动态管理、综合治理、利益导向三大机制，深入开展"两无"活动和打击"两非"工作，继续推进计生"转型升类"工程，力争今年消灭计生三类地区，稳定低生育水平。大力推行火葬，扭转殡改工作被动局面。发展社会福利和慈善事业。完善救助管理机制。加强军政军民团结，做好双拥工作。加强国防教育和国家安全教育，进一步提高民兵预备役建设水平。完成"十二五"规划及各专项规划编制工作。促进审计、统计、法制、人防、气象、防震、台务、港澳、妇女儿童、老龄、残联、文化广播电视、新闻出版、民族宗教、外事侨务、档案修志等工作全面发展。

　　十、以治理软环境为抓手，加快推进服务型政府建设

　　推进民主法制建设。切实加强民主政治建设，完善人大代表、政协委员反映民意的机制，严格按照法定权限和程序履行职责。认真落实市人大及其常委会各项决议决定，坚持向人大及其常委会报告工作，主动加强同政协的联系并向其通报工作，自觉接受监督。提高议案、建议和提案办理质量。坚持原则，依法办事，切实维护人民群众的利益。完善民主党派、无党派人士建言献策"直通车"制度。积极推进政务公开、村务公开、厂务公开，扩大基层民主。全面贯彻《国务院关于加强市县政府依法行政的决定》，加快推进依法治市进程，加强国家行政机关、司法机关队伍权威性建设，坚决落实依法行政、公正司法和严格执法。加强法制宣传教育，提高公民的法律素质。

　　切实维护社会安全。进一步完善大平安体系，深入开展"强综治、创平安、促维稳"活动，及时将社会矛盾纠纷化解在萌芽状态；开展社会治安综合治理和治安专项治理，切实加强反走私综合治理工作，促进社会秩序持续稳定向好；切实健全应急管理机制，提高预防和处置突发事件能力，确保亚锦赛、亚运会分赛区、惠州恳亲会的绝对安全；全面加强产品、食品、药品质量安全工作，强化监管，构建保障产品、食品、药品质量安全的长效机制；坚持安全发展，扎实推进各项专项整治，加强消防、道路交通、水上交通安全工作，坚决防范和遏制重特大安全事故发生。做好防灾减灾工作。

　　加强政府自身建设。加大力度推进"权威、责任、效能、廉洁"政府建设。进一步完善岗位目标责任制、首问责任制、服务承诺制和限时办结制等制度，把落实责任贯穿于政府工作的全过程，全面贯彻《广东省市厅级党政领导班子和领导干部落实科学发展观评价指标体系及评价办法》，按照《汕尾市人民政府部门行政首长问责暂行办法》以及《汕尾市领导干部重点工作分工责任制考核办法》，进一步建立完善对县（市、区）和市直单位的考核体系，加强检查督促，严肃查处行政不作为和乱作为，推动工作责任的落实，提高政府的执行力和公信力。推进电子政务建设，及时把政府决策、服务程序、办事方法向社会公布。建立和完善重大问题集体决策制度，提高决策的透明度和公众参与度。及时受理和查处群众投诉的问题。认真落实反腐倡廉建设各项规定。加强对领导干部、人财物管理使用、关键岗位的监督和制约，强化行政监察和审计监督，严防失职渎职、违法犯罪现象发生。完善政府采购、项目招投标等制度，堵塞漏洞。加强机关作风建设，开展机关作风暗访工作，解决办事难、"吃、拿、卡、要"等问题。加强对公务员的教育、培训和管理。

　　各位代表！2010年工作任务光荣而艰巨。让我们紧密地团结在以胡锦涛同志为总书记的党中央周围，在市委的正确领导下，振奋精神，坚定信心，齐心协力，开拓创新，转变作风，真抓实干，为全面实现"十一五"规划目标任务，把汕尾建设成为融入珠三角桥头堡和宜居宜业的现代化滨海新城而努力奋斗！
